# Peroxiredoxin 2 is associated with colorectal cancer progression and poor survival of patients

**DOI:** 10.18632/oncotarget.14801

**Published:** 2017-01-24

**Authors:** LingLong Peng, Rong Wang, JingKun Shang, YongFu Xiong, ZhongXue Fu

**Affiliations:** ^1^ Department of Gastrointestinal Surgery, The First Affiliated Hospital of Chongqing Medical University, Chongqing, 400014, China

**Keywords:** colorectal cancer, peroxiredoxin 2, antioxidant enzyme, prognosis

## Abstract

The present study was to investigate the clinical significance of peroxiredoxin 2 (PRDX2), an oncoenzyme, in the development and progression of colorectal cancer(CRC).We found levels of PRDX2 mRNA and protein were higher in CRC cell lines than in normal human colonic epithelial cells. PRDX2 expression was significantly up-regulated in CRC lesions compared with that in the adjacent noncancerous tissues. CRC tissues from 148 of 226 (65.5%) patients revealed high level of PRDX2 protein expression in contrast to only 13 of 226 (5.8%) PRDX2 strong staining cases in the adjacent noncancerous tissues. Increased expression of PRDX2 protein was significantly associated with poor tumor differentiation (*p* = 0.001), advanced local invasion (*p* = 0.046), increased lymph node metastasis (*p* = 0.008), and advanced TNM stage (*p* = 0.020). Patients with higher PRDX2 expression had a significantly shorter disease-free survival and worse disease-specific survival than those with low expression. Importantly, PRDX2 up-regulation was an independent prognostic indicator for stage I–III, early stage (stage I-II) and advanced stage (stage III) patients. In conclusion, our findings suggest PRDX2 up-regulation correlates with tumor progression and could serve as a useful marker for the prognosis of CRC.

## INTRODUCTION

Colorectal cancer (CRC) is a common malignant disease with high morbidity and mortality [1]. CRC not only is prevalent in the developed countries but also shows a trend of increase in the developing countries including china [2, 3]. Although the advanced treatment approaches were used for CRC, the prognosis of CRC is still not ideal [4]. At present, the clinical stage of CRC is the main indicator to assess the prognosis of patients, however, clinical outcome differs greatly even among patients of the same tumor–node–metastasis (TNM) stage [5]. Therefore, it is necessary to seek a significative and useful biomarker for the prognosis prediction of CRC patients.

Peroxiredoxin2 (PRDX2) is a typical 2-Cys thioredoxin peroxidase, which widely distributed in various tissues and cells. PRDX2 has been identified as an antioxidant enzymes that balance reactive oxygen species (ROS) and cytokine-induced peroxide levels using its thioredoxin as an intermediate electron donor [6]. Many researches have demonstrated that PRDX2 play an important part in various biological functions, such as the protection effects for intracellular lipids and proteins [7] and the mediation role for cellular signaling pathways associated with cell proliferation, apoptosis and differentiation [8–11]. In addition, over-expression of PRDX2 has been reported in various cancer tissues and cells, which is essential for tumor maintenance and survival by protecting cells against ROS injury and apoptosis [12–15]. As the hyperproliferative property of tumor cells is closely related to the increased production of intracellular ROS [16], Thus, the increased expression of PRDX2, as a scavenger of ROS in cancer cells, is beneficial for the survival and growth of tumor cell. Moreover, several studies have reported down-regulation of PRDX2 expression had a good therapeutic effect for cancer. For example, inhibiting PRDX2 expression sensitized head and neck cancer cells to radiation [17] and gastric carcinoma cells to cisplatin [18]. Moreover, PRDX2 knockdown augmented H_2_O_2_-induced cell death in hepatocellular carcinoma SMMC-7721 cells through enhancing ROS generation in response to H_2_O_2_, whereas PRDX2 over-expression exhibited opposite effects [12]. Furthermore, inhibiting PRDX2 expression decreased the growth of breast cancer metastatic cells in lungs [19]. Take together, these findings suggest that PRDX2 is closely associated with the proliferation, metastasis, radio-resistance and drug-resistance of cancer.

The tumor promoting role of PRDX2 in CRC was firstly reported by our research group. We firstly reported that the PRDX2 expression was up-regulated in colorectal tumor tissues in comparison with the noncancerous tissues adjacent to CRC [13]. Later, our another research showed that inhibiting PRDX2 expression augmented apoptosis, decreased cell growth, and increased endogenous ROS production through down-regulating Wnt/beta-catenin signaling pathway [20]. In addition, down-regulation PRDX2 expression inhibited VEGF mimicry formation of HCT116 cells through targeting VEGFR2 activation in colorectal carcinoma [21]. However, the clinical implication of the protein expression of PRDX2 in CRC has not been reported. Thus, we aimed to identify and confirm the expression of PRDX2 in CRC by several lines of evidence, and further investigate the prognostic value of PRDX2 in CRC using a large number of colorectal cancer tissue samples.

## RESULTS

### Up-regulation of PRDX2 in colorectal cancer cell lines

**Figure 1:**
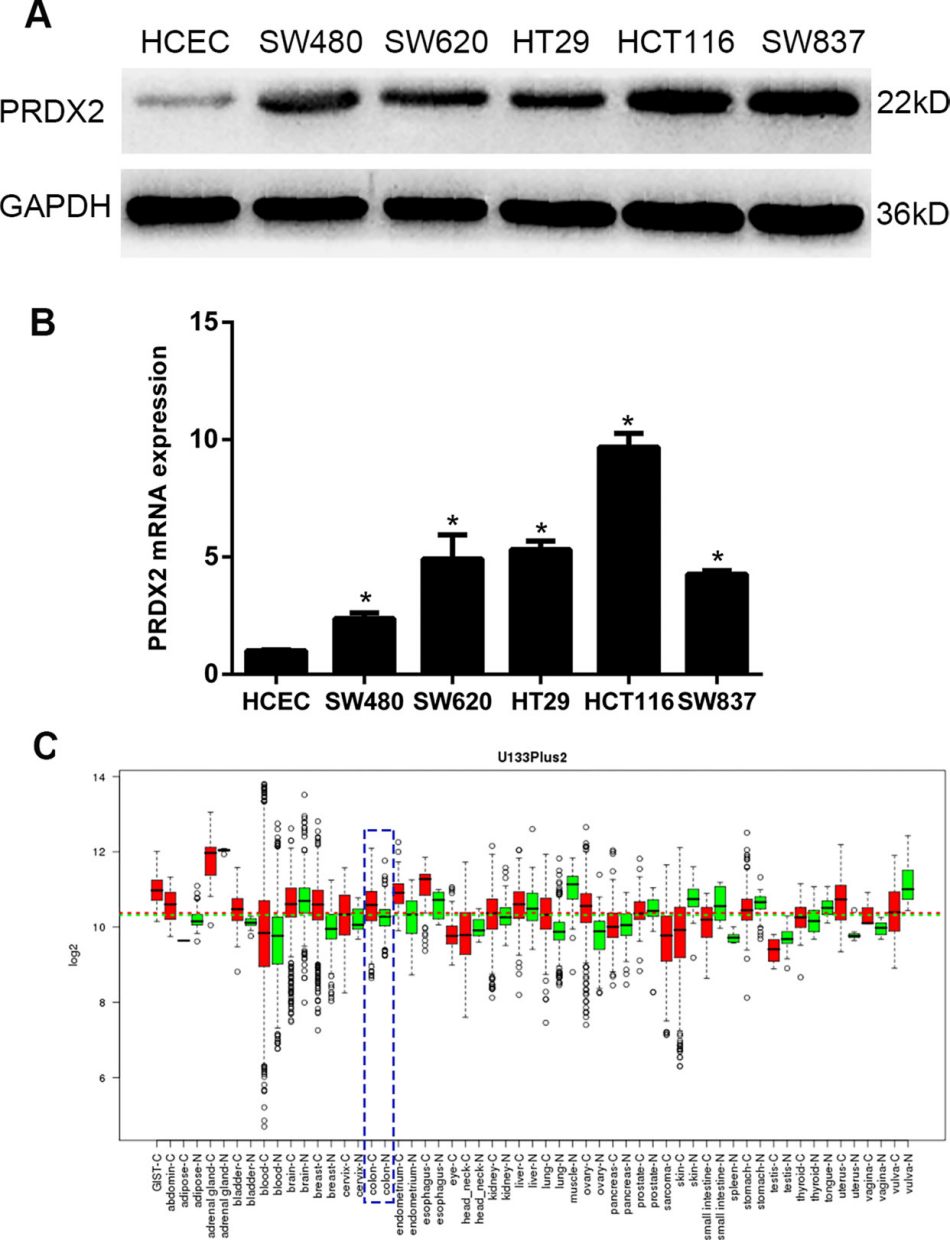
Expression analysis of PRDX2 protein and mRNA in HCEC and colorectal cancer cell lines by quantitative real-time reverse transcription-PCR (qRT-PCR) and Western blotting (**A**) Expression of PRDX2 protein in HCEC and cultured colorectal cancer cell lines SW480, SW620, HT29, HCT116, and SW837. (**B**) Expression of PRDX2 mRNA in HCEC and cultured colorectal cancer cell lines. Data represent the mean ± SD of three experiments. (*p < 0.01 vs HCEC). (**C**) PRDX2 mRNA expression in various types of cancer was searched in the GENT database (http://medical-genomics.kribb.re.kr/GENT/). Boxes represent the median and the 25th and 75th percentiles; dots represent outliers. Red boxes represent tumour tissues; green boxes represent normal tissues. Red and green dashed lines represent the average value of all tumour and normal tissues, respectively. The asterisk indicates the significant increase of PRDX2 expression in colon tumours compared with normal tissues. PRDX2 mRNA expression of colon tissue: blue dotted lines.

To determine PRDX2 protein expression, Western blotting analysis was conducted on protein samples derived from normal human colonic epithelial cells (HCEC) and several colorectal cancer cell lines. All cancer cell lines expressed high levels of PRDX2 protein compared with the HCEC (Figure 1A). To investigate whether PRDX2 up-regulation was at the transcription level, mRNA of PRDX2 in colonic cancer cell lines was quantified using qRT-PCR analysis. We found that all colorectal cancer cell lines revealed higher PRDX2 mRNA expression compared with those in HCEC (Figure 1B).

### Expression of PRDX2 is up-regulated in colorectal cancer lesions

**Figure 2:**
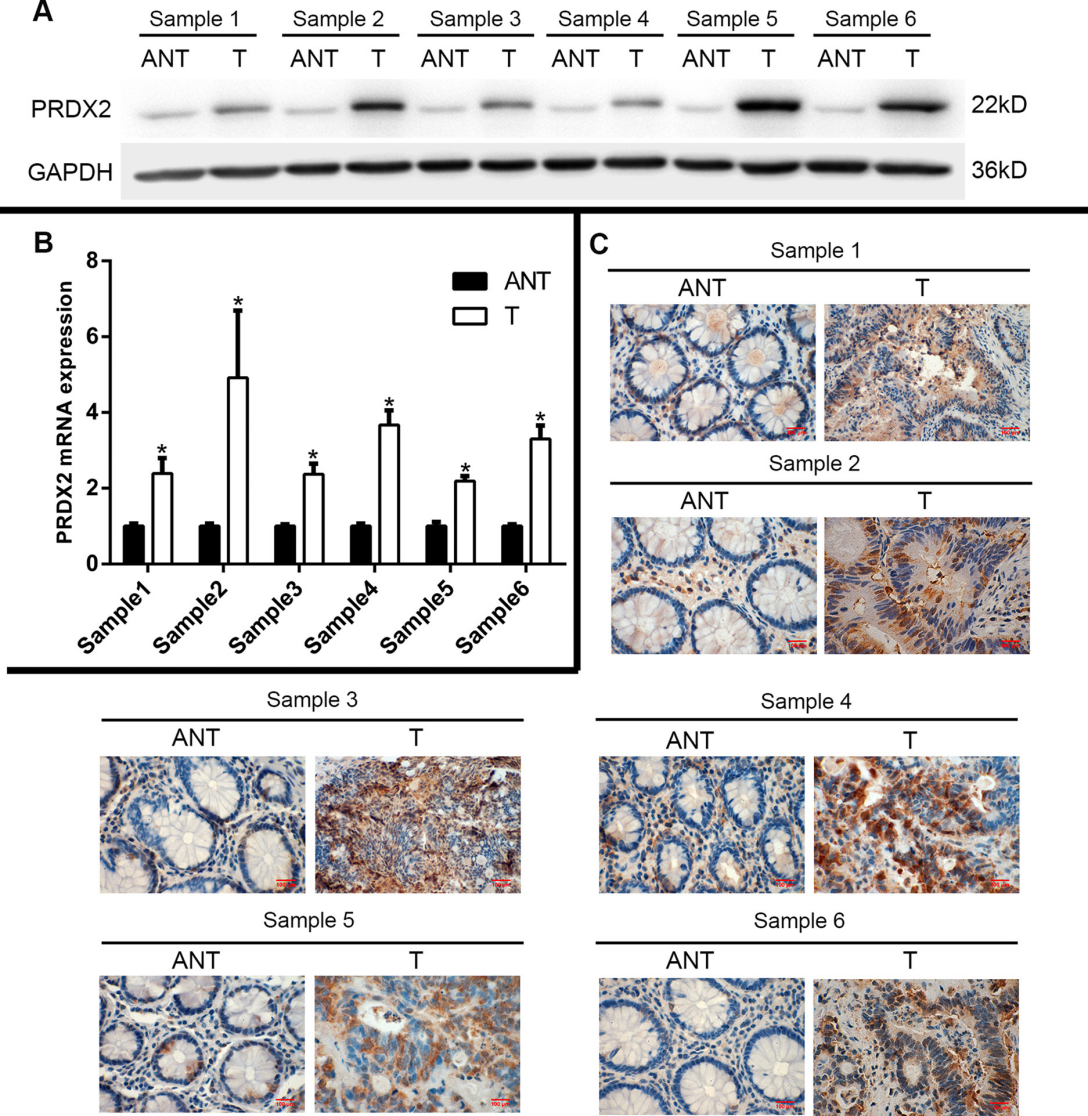
Expression of PRDX2 is elevated in primary colorectal tumors compared with human colorectal tumor-adjacent tissues (**A**) Expression of PRDX2 protein in each of the primary colorectal tumors and colorectal adjacent noncancerous tissues paired from the same patient by Western blotting, GAPDH was used as loading control. (**B**) Expression of PRDX2 mRNA in each of the primary colorectal tumors (T) and colorectal adjacent noncancerous tissues (ANT) paired from the same patient by quantitative real-time reverse transcription-PCR (qRT-PCR). (**C**) PRDX2 expression level was up-regulated in the primary colorectal tumor compared with the paired colorectal adjacent noncancerous tissues from the same patient, as examined by immunohistochemistry. Data represent the mean ± SD of three experiments. (*p < 0.01 vs ANT).

In the GENT database, PRDX2 is up-regulated in colorectal cancers compared with corresponding normal tissues (Figure 1C). To determine whether PRDX2 up-regulation in colorectal cancer cell lines could clinically correlate with CRC progression, Western blotting and qRT-PCR analysis were done in six matched colorectal cancer lesions and noncancerous tissues adjacent to colorectal cancer lesions. The protein and mRNA levels of PRDX2 were found to be significantly over-expressed in all six examined human primary colorectal cancer samples compared with adjacent noncancerous tissues (Figure 2A and 2B). In addition to the Western blotting, six tumor samples were further detected for PRDX2 expression by immunohistochemical analysis. In agreement with the result of Western blotting assay, immunohistochemical analysis also showed PRDX2 over-expression in all six tumors in comparison with the noncancerous tissues adjacent to colorectal adenomas (Figure 2C).

### PRDX2 expression is significantly and frequently up-regulated in archived colorectal cancer tissue samples

**Figure 3:**
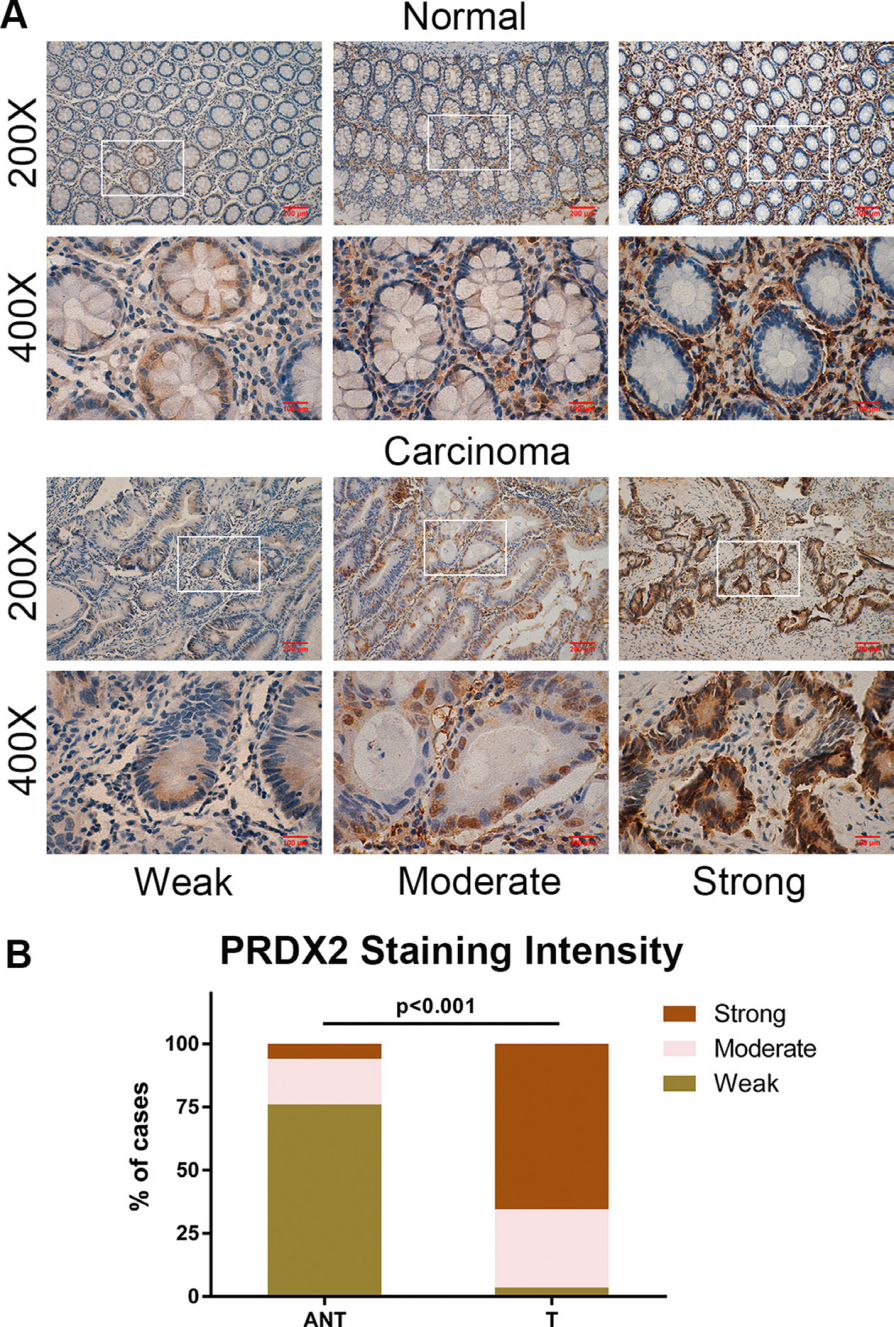
PRDX2 is frequently up-regulated in CRC (**A**) Representative immunohistochemical expression patterns of PRDX2 in 226 paired human primary colorectal cancer tissues and corresponding adjacent normal mucosa specimens are shown. (**B**) Percentage of cases with different staining intensity of PRDX2 in the tumor or adjacent normal tissues in the study cohort.

To further examine whether PRDX2 protein up-regulation is linked to clinical progression of colorectal cancer, immunohistochemical analysis was performed in 226 paired CRC specimens. In Figure 3A, PRDX2 expression was mainly located in the nucleus and cytoplasm of cancer cells. In these specimens, 65.5% (148/226) of the tumor tissues showed strong staining, 31.0% (70/226) of the tumor tissues had a moderate staining, 3.5% (8/226) of the cases indicated weak staining and no cases with a negative staining. In contrast, none of the matched non-cancerous cases showed negative staining, 76.1% (172/226) of the non-cancerous tissues showed weak staining, 18.1% (41/226) of the cases indicated moderate staining and only 5.8% (13/226) of the cases had a strong staining of PRDX2 (*p* < 0.001, Figure 3A and 3B). Taken together, these observations suggest that PRDX2 protein expression was significantly and frequently up-regulated in CRC and high levels of PRDX2 expression are associated with clinical development of CRC.

### Association of PRDX2 expression with clinicopathologic features

226 CRC patients were divided into low and high PRDX2 expression subdivisions as described in the Methods. High PRDX2 expression was significantly related with local invasion (*p* = 0.046), TNM stage of CRC (*p* = 0.020), tumor differentiation (*p* = 0.001), and lymph node metastasis (*p* = 0.008) (Table 1). However, no significant associations was observed between PRDX2 expression and tumor location (*p* = 0.604), serum CEA level (*p* = 0.629), preoperative bowel obstruction or perforation (*p* = 0.644), age (*p* = 0.412), sex (*p* = 0.833), or tumor size (*p* = 0.471).

**Table 1:** Association between PRDX2 expression and clinicopathologic characteristics of CRC patients in the study cohort

Characteristics	No. of patients (%)	PRDX2 expression		*P* value
		Low (%)	High (%)	
	(*n* = 226)	(*n* = 97)	(*n* = 129)	
**Age (years)**				0.412
< 60	84(37.2%)	39(40.2%)	45(34.9%)	
≥ 60	142(62.8%)	58(59.8%)	84(65.1%)	
**Sex**				0.833
Female	109(48.2%)	46(47.4%)	63(48.8%)	
Male	117(51.8%)	51(52.6%)	66(51.2%)	
**Tumor location**				
Colon	119(52.7%)	53(54.6%)	66(51.2%)	0.604
Rectum	107(47.3%)	44(45.4%)	63(48.8%)	
**Tumor size (cm)**				0.471
< 5	90(39.8%)	36(37.1%)	54(41.9%)	
≥ 5	136(60.2%)	61(62.9%)	75(58.1%)	
**Bowel obstruction/perforation**				0.644
No	216(95.6%)	92(94.8%)	124(96.1%)	
Yes	10(4.4%)	5(5.2%)	5(3.9%)	
**Differentiation grade**				0.001
Well	18(8.0%)	12(12.4%)	6(4.7%)	
Moderate	144(63.7%)	69(71.1%)	75(58.1%)	
Poor	64(28.3%)	16(16.5%)	48(37.2%)	
**Serum CEA level (ng/mL)**				0.629
< 10	103(45.6%)	46(47.4%)	57(44.2%)	
≥ 10	123(54.4%)	51(52.6%)	72(55.8%)	
**Local invasion**				0.046
T1–T2	44(19.5%)	13(13.4%)	31(24.0%)	
T3–T4	182(80.5%)	84(86.6%)	98(76.0%)	
**Lymph node metastasis**				0.008
N0	120(53.1%)	59(60.8%)	61(47.3%)	
N1	84(37.2%)	35(36.1%)	49(38.0%)	
N2	22(9.7%)	3(3.1%)	19(14.7%)	
**TNM stage**				0.020
I	17(7.5%)	12(12.4%)	5(3.9%)	
II	103(45.6%)	47(48.5%)	56(43.4%)	
III	106(46.9%)	38(39.2%)	68(52.7%)	

Abbreviations: CEA, carcinoembryonic antigen; PRDX2, peroxiredoxin 2; TNM, tumor-node-metastasis.

### The prognostic value of PRDX2 in stage I-III, early stage (stage I-II) and advanced stage (stage III) patients

**Figure 4:**
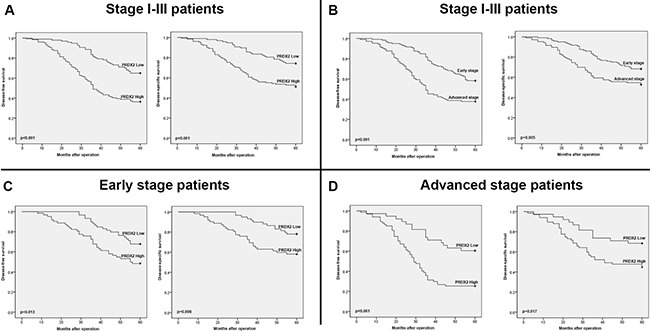
Kaplan-Meier survival analysis (**A**) Kaplan-Meier curves for disease-free survival and disease-specific survival of stage I-III CRC patients in the study cohort according to PRDX2 expression status (high or low expression). (**B**) Kaplan-Meier curves for disease-free survival and disease-specific survival of stage I-III CRC patients in the study cohort according to TNM stage (TNMI - II stage or TNMIII stage). (**C**) Kaplan-Meier curves for disease-free survival and disease-specific survival of CRC patients with early stage (stage I-II) tumors in the study cohort according to PRDX2 expression status (high or low expression). (**D**) Kaplan-Meier curves for disease-free survival and disease-specific survival of CRC patients with advanced stage (stage III) tumors in the study cohort according to PRDX2 expression status (high or low expression). The *p-value* was determined using the log-rank test.

A longer DFS and DSS were observed in patients (stage I-III) with the low expression of PRDX2 than those patients with the high PRDX2 expression (*p* < 0.001 and *p* < 0.001, respectively; Figure 4A). The cumulative 5-year DFS and DSS rate was 64.9% (95% confidence interval (95% CI) = 55.4%–74.3%) and 74.2% (95% CI = 65.6%–88.2%) in the low PRDX2 group, while it was only 36.4% (95% CI = 28.2%–44.6%) and 51.2% (95% CI = 42.6%–59.8%) in the high PRDX2 group, respectively.

To investigate whether PRDX2 expression indicates an independent prognostic biomarker in CRC, Cox regression analysis was used to analyze the effect of each variable on survival. Univariate analysis results showed DFS and DSS were related with the expression of PRDX2, TNM stage of CRC, tumor differentiation grade and patient age (Table 2). And then, those significant variables were further evaluated by multivariate analysis, which showed that the expression of PRDX2 (hazard ratio (HR) = 2.322, 95% CI = 1.536–3.509, *p* < 0.001) and TNM stage (HR = 1.933, 95% CI = 1.331–2.807, *p* = 0.001) were independent prognostic indicators for DFS (Table 2). Moreover, multivariate analysis showed that the independent prognostic variables for DSS included patient age (HR = 1.652, 95% CI=1.030–2.649, *p* = 0.037), PRDX2 expression (HR = 2.046, 95% CI = 1.270–3.296, *p* = 0.003), tumor differentiation grade (HR = 1.671, 95% CI = 1.079–2.589, *p* = 0.022), and TNM stage of CRC (HR = 1.578, 95% CI = 1.030–2.417, *p* = 0.036) (Table 2).

**Table 2:** Univariate and multivariate analyses of prognostic factors for disease-free survival or disease-specific survival of stage I-III CRC patients in the study cohort

Variables	Univariate analysis^a^			Multivariate analysis		
	HR	95 % CI	*P* value	HR	95 % CI	*P* value
**Disease-free survival**						
Age (≥ 60/< 60 years)	1.495	1.008–2.216	**0.045**	1.350	0.907–2.010	0.139
Sex(male/female)	1.014	0.705–1.460	0.939			
Tumor location(rectum/colon)	1.141	0.793–1.642	0.478			
Tumor size (≥ 5/< 5 cm)	1.025	0.707–1.487	0.895			
Bowel obstruction/perforation (yes/no)	0.955	0.390–2.340	0.920			
Differentiation grade (poor/well + moderate)	1.807	1.236–2.643	**0.002**	1.402	0.949–2.070	0.090
Local invasion (T3–T4/T1–T2)	1.074	0.675–1.708	0.764			
Serum CEA level (≥ 10/ < 10 ng/mL)	1.002	0.695–1.444	0.991			
TNM stage (III/I + II)	2.098	1.451–3.034	**< 0.001**	1.933	1.331–2.807	**0.001**
PRDX2 expression (high/low)	2.575	1.724–3.845	**< 0.001**	2.322	1.536–3.509	**< 0.001**
**Disease-specific survival**						
Age (≥ 60/< 60 years)	1.752	1.096–2.801	**0.019**	1.652	1.030–2.649	**0.037**
Sex (male/female)	1.014	0.668–1.540	0.948			
Tumor location (colon/rectum)	1.084	0.714–1.647	0.704			
Tumor size (≥ 5/< 5 cm)	0.852	0.559–1.299	0.457			
Bowel obstruction/perforation (yes/no)	0.978	0.359–2.668	0.966			
Differentiation grade (poor/well + moderate)	2.019	1.316–3.097	**0.001**	1.671	1.079–2.589	**0.022**
Local invasion (T3–T4/T1–T2)	1.229	0.705–2.142	0.468			
Serum CEA level (≥ 10/ < 10 ng/mL)	1.017	0.668–1.547	0.939			
TNM stage (III/I + II)	1.809	1.186–2.760	**0.006**	1.578	1.030–2.417	**0.036**
PRDX2 expression (high/low)	2.388	1.502–3.798	**< 0.001**	2.046	1.270–3.296	**0.003**

Abbreviations: CEA, carcinoembryonic antigen; CI, confidence interval; HR, hazard ratio; PRDX2, peroxiredoxin 2; TNM, tumor-node-metastasis.

^a^ In univariate analyses, log-rank tests were conducted.

^b^ In the multivariate Cox proportional hazard model, only variables with *P* < 0.05 in univariate analysis were included and the “enter method” was applied.

The correlation of TNM stage with clinical outcome was also analyzed. A shorter and worse DFS and DSS were observed in the advanced stage (stage III) patients than the early stage (stages I-II) patients (*p* < 0.001 and *p* = 0.005, respectively; Figure 4B).

Based on the above results, to further investigate the prognostic significance of PRDX2 in CRC patients with same stage classification, we divided patients according to TNM stage and performed survival analysis based on PRDX2 expression. Remarkably, high PRDX2 expression predicted a worse DFS and DSS not only in early stage (stage I-II) patients (*p* = 0.013 and *p* = 0.008, respectively; Figure 4C), but also in advanced stage (stage III) patients (*p* < 0.001 and *p* = 0.017, respectively; Figure 4D).

The independent prognostic value of PRDX2 expression on survival according to TNM stage was further assessed by Cox regression model. For early stage (stage I-II) patients, univariate analysis showed that PRDX2 expression, tumor differentiation grade and patient age were significantly related with DFS and DSS (Table 3). Those significant parameters were further evaluated through multivariate analyses, and the results showed patient age (DSS, HR = 2.235, 95% CI = 1.083–4.613, *p* = 0.030) and high PRDX2 expression (DFS, HR=1.856, 95% CI = 1.016–3.390, *p* = 0.044; DSS, HR = 2.137, 95% CI = 1.062–4.299, *p* = 0.033) remained independent prognostic indicators of poor DFS and DSS in early stage patients (Table 3).

**Table 3:** Univariate and multivariate analyses of prognostic factors for disease-free survival or disease-specific survival of patients with early stage (stage I-II) tumors in the study cohort

Variables	Univariate analysis^a^			Multivariate analysis^b^		
	HR	95 % CI	*P* value	HR	95 % CI	*P* value
**Disease-free survival**						
Age (≥ 60/< 60 years)	1.812	1.002–3.275	0.049	1.780	0.980–3.232	0.058
Sex (male/female)	0.722	0.414–1.256	0.249			
Tumor location (rectum/colon)	1.576	0.905–2.745	0.108			
Tumor size (≥ 5/< 5 cm)	0.860	0.496–1.494	0.593			
Bowel obstruction/perforation (yes/no)	1.294	0.315–5.325	0.721			
Differentiation grade (poor/well + moderate)	1.857	1.035–3.332	0.038	1.392	0.747–2.594	0.298
Local invasion (T3–T4/T1–T2)	1.255	0.644–2.447	0.505			
Serum CEA level (≥ 10/ < 10 ng/mL)	1.167	0.674–2.021	0.581			
PRDX2 expression(high/low)	2.018	1.143–3.561	0.015	1.856	1.016–3.390	0.044
**Disease-specific survival**						
Age (≥ 60/< 60 years)	2.300	1.120–4.721	0.023	2.235	1.083–4.613	0.030
Sex (male/female)	0.737	0.391–1.389	0.346			
Tumor location (colon/rectum)	1.330	0.708–2.496	0.375			
Tumor size (≥ 5/< 5 cm)	0.578	0.308–1.085	0.088			
Bowel obstruction/perforation (yes/no)	1.713	0.412–7.112	0.459			
Differentiation grade (poor/well + moderate)	2.190	1.146–4.183	0.018	1.585	0.801–3.137	0.186
Local invasion (T3–T4/T1–T2)	1.316	0.605–2.864	0.488			
Serum CEA level (≥ 10/ < 10 ng/mL)	1.298	0.692–2.437	0.416			
PRDX2 expression (high/low)	2.377	1.221–4.629	0.011	2.137	1.062–4.299	0.033

Abbreviations: CEA, carcinoembryonic antigen; CI, confidence interval; HR, hazard ratio; PRDX2, peroxiredoxin 2; TNM, tumor-node-metastasis.

^a^ In univariate analyses, log-rank tests were conducted.

^b^ In the multivariate Cox proportional hazard model, only variables with *P* < 0.05 in univariate analysis were included and the “enter method” was applied.

Furthermore, in univariate analysis of advanced stage (stage III) patients, those who with high PRDX2 expression and poor differentiation grade had a shorter DFS and DSS (Table 4). However, only high expression of PRDX2 protein (DFS, HR = 2.750, 95% CI = 1.525–4.961, *p* = 0.001; DSS, HR = 1.990, 95% CI = 1.027–3.854, *p* = 0.041) was an independent factor of a poor prognosis for advanced stage tumors by using Cox multivariate analysis (Table 4). Take together, These results showed that in addition to over-expression of PRDX2 in CRC tissues significantly predicted poor DFS and DSS, high PRDX2 expression also was an independent predictor of poor prognosis in stage I-III, early stage (stage I-II) and advanced stage (stage III) patients.

**Table 4:** Univariate and multivariate analyses of prognostic factors for disease-free survival or disease-specific survival of patients with advanced stage (stage III) tumors in the study cohort

Variables	Univariate analysis^a^			Multivariate analysis^b^		
	HR	95 % CI	*P* value	HR	95 % CI	*P* value
Disease-free survival						
Age (≥ 60/< 60 years)	1.029	0.608–1.744	0.915			
Sex (male/female)	1.311	0.802–2.144	0.280			
Tumor location (rectum/colon)	0.868	0.533–1.413	0.569			
Tumor size (≥ 5/< 5 cm)	1.104	0.664–1.836	0.702			
Bowel obstruction/perforation (yes/no)	0.691	0.217–2.201	0.531			
Differentiation grade (poor/well + moderate)	1.671	1.012–2.758	0.045	1.392	0.837–2.313	0.202
Local invasion (T3–T4/T1–T2)	0.669	0.349–1.281	0.225			
Serum CEA level (≥ 10/ < 10 ng/mL)	0.716	0.438–1.172	0.184			
PRDX2 expression (high/low)	2.920	1.633–5.224	< 0.001	2.750	1.525–4.961	0.001
Disease-specific survival						
Age (≥ 60/< 60 years)	1.196	0.643–2.223	0.572			
Sex (male/female)	1.274	0.723–2.245	0.402			
Tumor location (colon/rectum)	0.944	0.539–1.652	0.839			
Tumor size (≥ 5/< 5 cm)	1.104	0.613–1.988	0.741			
Bowel obstruction/perforation (yes/no)	0.592	0.144–2.437	0.468			
Differentiation grade (poor/well + moderate)	1.824	1.031–3.227	0.039	1.625	0.911–2.898	0.100
Local invasion (T3–T4/T1–T2)	0.905	0.406–2.016	0.807			
Serum CEA level (≥ 10/ < 10 ng/mL)	0.724	0.412–1.273	0.260			
PRDX2 expression (high/low)	2.163	1.127–4.151	0.020	1.990	1.027–3.854	0.041

Abbreviations: CEA, carcinoembryonic antigen; CI, confidence interval; HR, hazard ratio; PRDX2, peroxiredoxin 2; TNM, tumor-node-metastasis.

^a^ In univariate analyses, log-rank tests were conducted.

^b^ In the multivariate Cox proportional hazard model, only variables with *P* < 0.05 in univariate analysis were included and the “enter method” was applied.

## DISCUSSION

Peroxiredoxins (PRDXs) are a ubiquitous family of antioxidant proteins, which balance cellular reactive oxygen species (ROS) and cytokine-induced peroxide levels, and involved in regulating various biological behavior including cell signaling transduction and metabolism [23, 24]. A series of researches have concluded that PRDXs are involved in some particular pathological conditions such as cancer, inflammatory diseases and neurodegenerative diseases [25]. In particular, PRDX isoforms were regarded as good therapeutic targets in various type of cancers including prostate cancer [26], colorectal cancer [13, 20, 21], glioblastoma [27], lung cancer [28] and ovarian cancer [29, 30]. Currently, a total of six PRDXs isozymes (PRDX 1–6) have been identified in mammalian systems, and PRDX2 belongs to typical 2-Cys group with two conserved cysteine residues [31]. Functional studies demonstrated that PRDX2 play a tumor promoting effect in various cancers including colorectal carcinoma. Shiota et al. [32] demonstrated that PRDX2 increased the growth and castration resistance of prostate cancer by regulating the androgen/androgen receptor signaling pathway. Kwon et al. [33] reported that PRDX2 maintains cancer stem cells selfrenewal through VEGF signaling and protects oxidative inactivation of VEGFR2 in hepatocellular carcinoma cells. Stresing et al. [19] concluded that inhibiting PRDX2 expression decreased the growth of breast cancer metastatic cells in lungs by specifically regulating the oxidative and metabolic stress response. Beside that, our previous studies also showed that PRDX2 knockdown in CRC cell lines augmented apoptosis, decreased cell growth, and increased endogenous ROS production through down-regulating Wnt/beta-catenin signaling pathway [20]. These data indicate that PRDX2 is closely related with the development and progression of various cancers including colorectal cancer.

Considering the multiple effects of PRDX2 in carcinoma pathobiology, its prognostic implication in malignant diseases has triggered widespread attention. Lomnytska et al. [34] demonstrated that detection of the alteration of PRDX2 expression may aid current cytological and pathological diagnostics and evaluation of prognosis in squamous cervical cancer precursor lesions. In addition, a recent study identified that PRDX2 is a predictive indicator for the induction chemotherapy response in osteosarcoma using proteomics study of open biopsy samples [35]. Moreover, by using systematic profiling of DNA methylation at CpG island promoters of pathways relevant to ovarian carcinogenesis, Dai et al. have identified the DNA methylation biomarker-PRDX2 that give rise to a methylation index capable of predicting progression free survival in ovarian cancer independently from known clinical prognostic feature [36]. Currently, only our previous studies tested PRDX2 expression in CRC. In our previous study, all six PRDXs isoforms have been examined in paired cancer and non-cancer tissues by Immunohistochemistry and Western blotting, and the results showed that stage III patients and those cases with lymph node metastasis has a higher expression of PRDX2 [37]. Interestingly, our another previous study also indicated that over-expression PRDX2 in CRC was strongly associated with TNM stage of CRC and distant metastasis [13]. However, only 32 and 35 CRC tissue samples were detected in our previous studies, a large enough samples were essential to assess the prognostic value of PRDX2 expression in CRC. More importantly, if PRDX2 was identified a useful marker for prognostic evaluation and guidance, it is clinically significant for CRC patients. These reasons prompted us to perform this study. In our current study, over-expression of PRDX2 in CRC was identified and confirmed by several lines of evidence, including assessment of PRDX2 mRNA and protein expression in CRC cell lines compared with those in HCEC, comparative determination of PRDX2 expressions in six matched CRC lesions and adjacent noncancerous tissues, and a clear demonstration of generally high level of PRDX2 expression in a relatively large number of 226 paired CRC specimens and noncancerous colorectal tissues. Moreover, our results showed that over-expression of PRDX2 was significantly related with local invasion, TNM stage of CRC, tumor differentiation, and lymph node metastasis. These findings further emphasized the tumor promoting effects of PRDX2 which related to the progression of CRC.

In our current study, we found that the high PRDX2 expression was significantly related with a worse DFS and DSS, and was an independent prognostic marker of poor clinical outcome in CRC. These findings were consistent with the study of Lomnytska et al., in that they reported those patients with the high PRDX2 expression has a shorter survival time than those who with a low PRDX2 expression in squamous cervical cancer [34]. Moreover, our current study reported that TNM stage of CRC was also an critical prognostic indicator, which consisted with the well established adverse prognostic effect of tumor TNM stage [38]. At present, the TNM staging system of the AJCC provides the most reliable guidelines for the prognostication and treatment of colorectal cancer. However, several multicenter and large sample researches suggest that TNM classification does not meet their expectations because of the limited advancement in their prognostic implication [39, 40]. For example, in the current edition of TNM7, tumor nodules that were determined not to be lymph node metastasis (LNM) are considered different from other lymph nodes related with the TNM staging process. Therefore, the number of tumor nodules not regarded as LNM does not affect the subdivisions within stage III; but the number of tumor nodules provide an important prognostic implication [41]. Besides that, clinical prognosis varies greatly in early stage CRC patients, although the factor of lymph node metastasis was not be considered in that stage tumors [4, 5, 10]. In the present study, our stage-based survival analysis showed high PRDX2 expression in CRC tissues had a poor DFS and DSS, and was an independent indicator for poor prognosis in early and advanced stage tumors, suggesting that PRDX2 can be as a novel indicator to divide patients with early and advanced stage tumors into distinct risk subdivisions.

In conclusion, our current study has revealed that PRDX2 expression was significantly and frequently up-regulated in CRC and was significantly related with disease progression. More importantly, in addition to high PRDX2 expression in CRC significantly predicted poor DFS and DSS, over-expression PRDX2 also was an independent unfavorable prognostic indicator in stage I-III, early stage (stage I-II) and advanced stage (stage III) patients. Our study has provided a basis for the development of a novel biomarker for the prognosis of CRC.

## MATERIALS AND METHODS

### Cell lines and antibodys

Primary cultures of human colonic epithelial cells (HCEC) were established from colonic biopsies taken during intestinal endoscopy and cultured. The colorectal cancer cell lines, including HCT116, HT29, SW480, SW620, and SW837, were obtained from the American Type Culture Collection and kept in our laboratory. These cell lines were cultured in RPMI 1640 medium (Gibco, Grand Island, NY) supplemented with 10% fetal bovine serum (FBS) (PAN, Germany) and 1% penicillin/streptomycin (Beyotime, Jiangsu, China) and maintained at 37°C and 5 % CO_2_ in a humidified atmosphere.

Rabbit monoclonal antibody to Peroxiredoxin 2[EPR5154] (ab109367) was obtained from Abcam PLC (Cambridge, MA, USA).

### Patients and specimens

Paired paraffin-embedded CRC and corresponding adjacent normal mucosa specimens from 226 stages I–III CRC patients who received curative surgery in The First Affiliated Hospital of Chongqing Medical University (Chongqing, China) from January 2010 to April 2011 were retrieved for immunohistochemistry. The study cohort consisted of patients with CRC as confirmed by pathological analysis. Detailed clinicopathologic characteristics of the patients are listed in Table 1, and Supplementary Table 1 and 2. Patient inclusion criteria included the following: (1) patients with a pathological diagnosis of colorectal cancer; (2) patients with a primary tumor without evidence of distant metastasis (TNM stage I–III); (3) patients who were treated primarily with surgery; (4) patients with no previous treatment; and (5) patients with complete clinicopathological data and available tissue specimens. Patients were excluded from the study cohorts with the following exclusion criteria: previously received any anti-cancer therapy; impaired heart, lung, liver, or kidney function; previous malignant disease; failure to undergo surgery and the inability to obtain pathological slices.

Six paired fresh-frozen CRC tissue samples and adjacent noncancerous tissues from the same patient were obtained from absolute curative surgery, which had been clinically and histopathologically diagnosed at the Departments of Gastrointestinal Surgery and Pathology, The First Affiliated Hospital, Chongqing Medical University. These samples were used for quantitative real-time reverse transcription-PCR (qRT-PCR), Western blotting and immunohistochemistry analysis.

### Follow-up

The follow-up period was defined as the interval from the date of surgery to the date of death or last follow-up. The latest follow-up was updated in June 2016. All patients were followed up regularly in the outpatient clinic every 3 months during the first year, every 6 months until the fifth year, and then annually. All included subjects had complete follow-up information until death or the latest follow-up date. At follow-up visits, all medical data regarding preoperative diagnosis, surgery, recurrence, clinical staging, adjuvant treatment, clinical follow-up, and cause of death were re-assessed and recorded to our database by a surgery specialist. The current vital status of each patient was reviewed by confirming deaths from the hospital's patient registry or, if uncertain, from the service of the China Population Register Centre. Disease-specific survival (DSS) was defined as the interval from the date of surgery to the date that the patient died of CRC. Patients alive at the end of follow-up were censored. Disease-free survival (DFS) was defined as the interval between the day that surgery was performed and the day that recurrence was first detected. If recurrence was not diagnosed, the date of death due to CRC or of last follow-up was used. TNM staging was classified according to the criteria proposed by the Standard American Joint Committee on Cancer (AJCC).

The study was approved by the ethics committee on human research at Chongqing Medical University. Informed and written consents were obtained from the patients or their relatives for the use of these clinical materials for research, which were performed in accordance with the Declaration of Helsinki of the World Medical Association.

### Quantitative real-time reverse-transcription PCR analysis

Total RNAs were isolated from cells and frozen specimens using the RNAiso plus reagent (Takara). RNA (1 μg) was reverse-transcribed with the PrimeScript™ RT reagent Kit and gDNA Eraser (Takara). All the reactions were performed in triplicate, and the GAPDH gene was used as the internal control. Primer sequences used for the amplification of human genes were as follows: Prdx2 (forward 5′-CAC CTG GCT TGG ATC AAC ACC-3′ and reverse 5′-CAG CAC GCC GTA ATC CTC AG-3′) and GAPDH (forward 5′-ACC ACA GTC CAT GCC ATC CAC-3′ and reverse 5′-TCC ACC ACC CTG TTG CTG TA-3′). The relative expression levels of mRNAs were calculated using the 2^-(ΔCt sample–ΔCt control)^ method.

### Western blot analysis

To analyze the expression levels of total PRDX2, tumor tissues and cells were rinsed with PBS and then lysed in Lysis Buffer containing 150 mM sodium chloride, 0.1 M Tris, 1 % Tween-20, 50 mM diet- hyldithiocarbamic acid, 1 mM ethylenediamine tetraacetic acid, and protease inhibitors at pH 8.0. The crude lysate was centrifuged at 12,000 rpm at 4°C for 20 min, and the clarified cell extract was used for immunoblotting. The protein concentration was determined with a BCA Protein Assay Kit (Beyotime Biotechnology, China), according to the manufacturer's instructions. Proteins were separated by sodium dodecyl dulfate polycrylamide gel electrophoresis (SDS-PAGE), transferred onto polyvinylidenefluoride (PVDF) membranes (Immobilon-P, Millipore, Germany), blocked with 5% skim milk in TBST (20 mM Tris-HCl, 150 mM NaCl, 0.1% Tween 20), and blotted with rabbit monoclonal anti-Prdx2 (1:1000) and polyclonal anti-GAPDH (1:1000) primary antibodies at 4°C overnight. After washing with TBS containing 0.1 % Tween 20, the membranes were incubated with the proper secondary antibody (1:5000) for 1 h. The antigen-antibody complexes were detected by chemiluminescence (Millipore, Germany). with a chemiluminescence detection system (VILBER FusionFX, France).

### Immunohistochemistry analysis

Immunohistochemistry of paraffin-embedded tissue sections was performed as described previously [20]. Briefly, paraffin-embedded tissue samples were sectioned at 5 μm for immunohistochemical analysis and mounted on polylysine-coated slides. Ten tissue sections were cut from each sample of both CRC and controls, and two tissue sections of each sample were randomly selected for immunohistochemical analysis. Tissue sections were deparaffinized in xylene and rehydrated in a graded series of ethanol before staining. The endogenous peroxidase activity was blocked with 3% hydrogen peroxide for 10 min. Antigens were retrieved with citrate buffer (10 mM, pH 6.0) for 15 min at 100°C in a microwave oven. After blocking, the sections were incubated with rabbit monoclonal anitibody to PRDX2 (1:1000) (Abcam, USA) overnight at 4°C, the slides were rinsed with PBS and secondary antibodies were applied for 30 min at room temperature. Peroxidases bound to the antibody complex were visualized by treatment with a 3,3-diaminobenzidine chromogenic substrate solution. Immunolabeled sections were dehydrated with graded ethanol and defatted in xylenes. The sections were then visualized using an Olympus BX51 microscope (Olympus, Japan) under bright-field illumination, and images were acquired with an Olympus DP70 camera (Olympus, Japan). Images were processed and Average Integrated Optical Density (AIOD) were obtained from 10 random 200x microscopic fields with image-pro plus version 6.0(Media Cybernetics, Bethesda, MD, USA).

The degree of immunostaining was reviewed and scored independently by two observers in a blinded manner without prior knowledge of the clinical data based on the proportion of positively stained tumor cells and intensity of staining. Tumor cell proportion was scored as follows: 0 (no positive tumor cells), 1 (< 10% positive tumor cells), 2 (10–35% positive tumor cells), 3 (35–70% positive tumor cells), and 4 (> 70% positive tumor cells). Staining intensity was graded according to the following criteria: 0 (no staining), 1 (weak staining = light yellow), 2 (moderate staining = yellow brown), and 3 (strong staining = brown). Staining index was calculated as the product of staining intensity score and the proportion of positive tumor cells. Using this method of assessment, we evaluated PRDX2 expression in paired colorectal cancer tissues and the adjacent noncancerous tissues by determining the staining index, with scores of 0, 1, 2, 3, 4, 6, 9, or 12. The cutoff value for high and low expression level was chosen based on a measure of heterogeneity with the log-rank test statistical analysis with respect to DSS [22]. An optimal cutoff value was identified: a staining index score of ≥ 6 was used to define tumors with high PRDX2 expression and a staining index score of ≤4 was used to indicate low PRDX2 expression. If there was a discrepancy in individual evaluations, then the cases were reevaluated together with other pathologists to reach a consensus.

### Statistical analysis

Continuous data are presented as mean±standard deviation (SD). Independent Student's t test was used for continuous variables. Mann–Whitney U test was used to compare PRDX2 levels between groups. Pearson chi-square test or Fisher exact test was used to analyze the relationship between PRDX2 expression and clinical features. Kaplan–Meier analysis with logrank test was used to compare patients’survival between subgroups. The effect of each variable on survival was determined by the Cox multivariate regression analysis. All statistical analyses were carried out using SPSS Version 17.0 for Windows (SPSS, Inc., Chicago, IL), and *p* values < 0.05 were considered to be statistically significant.

## SUPPLEMENTARY MATERIALS FIGURES AND TABLES




