# Supplementary material for: Peroxiredoxin 2 is associated with colorectal cancer progression and poor survival of patients
Source: Oncotarget. 2017 Jan 24;8(9):15057–70. doi: 10.18632/oncotarget.14801 (PMC5362467; doi:10.18632/oncotarget.14801)
Supplement: Supplementary file 1 [file oncotarget-08-15057-s001.pdf]

Peroxiredoxin 2 is associated with colorectal cancer progression and poor survival of patients

Supplementary Materials

Supplementary Table 1: The original data of this study. See Supplementary_Table_1

Supplementary Table 2: Distribution of continuous variables of the study cohort

Variable	Median	Mean ± SEM	Range	Percentile	
				25th	75th
Age (years)	64.0	63.0 ± 0.7	35–89	55.0	70.3
Tumor size (cm)	5.7	6.1 ± 0.2	1.5–14.6	4.4	7.2
Serum CEA level (ng/mL)	12.0	13.5 ± 0.7	0.8–55.2	6.7	16.8
DFS(months)	55.0	44.9 ± 1.2	2–60	29	60
DSS(months)	60.0	48.1 ± 1.1	2–60	35	60

Abbreviations: CEA, carcinoembryonic antigen; DFS, disease-free survival; DSS, disease-specific survival; SEM, standard error of mean.
